# Supplementary material for: From subcritical behavior to a correlation-induced transition in rumor models
Source: Nat Commun. 2022 Jun 1;13:3049. doi: 10.1038/s41467-022-30683-z (PMC9160067; doi:10.1038/s41467-022-30683-z)
Supplement: Supplementary file 1 — Supplementary Information [file 41467_2022_30683_MOESM1_ESM.pdf]

From subcritical behavior to a correlation-induced transition in rumor models: Supplementary Information

Guilherme Ferraz de Arruda,[1,*] Lucas G. S. Jeub,[1] Angélica S. Mata,[2] Francisco A. Rodrigues,[3] and Yamir Moreno[1,4,5]

[1] *ISI Foundation, Via Chisola 5, 10126 Torino, Italy*
[2] *Departamento de Física, Universidade Federal de Lavras, 37200-900, Lavras, Minas Gerais, Brazil*
[3] *Departamento de Matemática Aplicada e Estatística, Instituto de Ciências Matemáticas e de Computação, Universidade de São Paulo - Campus de São Carlos, Caixa Postal 668, 13560-970 São Carlos, SP, Brazil.*
[4] *Institute for Biocomputation and Physics of Complex Systems (BIFI), University of Zaragoza, Zaragoza 50009, Spain*
[5] *Department of Theoretical Physics, University of Zaragoza, Zaragoza 50009, Spain*

I. COMPLEMENTARY SIMULATION RESULTS

Here we present the simulation results that were used to obtain the figures in the main text. First, we show the finite-size scaling for power-law networks, $P(k) \sim k^{-\gamma}$, for different values of α. The values of λ_c are obtained using the Figs. 1,2 and 3 where the susceptibility in function of λ was calculated for different uncorrelated power-law network sizes. First, we observe that, as α increases, the critical point also increases. Additionally, for $\gamma = 2.25$, our experiments suggest that the critical point vanishes in the thermodynamic limit. On the other hand, for $\gamma = 3.5$, we observe that the critical point converges to a non-zero value. For $\gamma = 2.75$, from our experiments, a vanishing behavior is reasonable. As mentioned in the main text, this behavior is at odds with the behavior of the SIS, in which the power-law networks have a vanishing critical point for any value of γ [1–3]. On the other hand, it follows a similar pattern as for the SIRS model [4], contact process [5], the generalized SIS model with weighted infection rates [6] and also modified versions of the SIS model [7].

Complementary, in Fig. 4 we show the estimation of the critical points for the random regular cases presented in Fig. 3 in the main text.

II. COMPLEMENTARY ASYMPTOTIC ANALYSIS: SIRS MODEL

For the sake of comparison, here we present the asymptotic analysis for the SIRS model. In this model, we have three different states for each node: (i) susceptible (x_i), which are healthy individuals that can be infected, (ii) infected (y_i), that are individuals that are spreading the infection, or (iii) recovered (z_i), individuals that already got the infection and are immune to the pathogen. In this model, the infection occurs through the contact between infected and susceptible individuals, which happens with rate λ. Next, infected individuals can heal, turning into recovered individuals, which occurs with a rate γ. Finally, the immunity is also lost with time, modeled by a spontaneous process that turns recovered individuals into susceptible individuals again. This process has a rate δ. The mean field equations that describe this process are

$$\begin{cases} \frac{dx_i}{dt} = & \delta z_i - \lambda \sum_{k=1}^N \mathbf{A}_{ki} x_i y_k \\ \frac{dy_i}{dt} = & \lambda \sum_{k=1}^N \mathbf{A}_{ki} x_i y_k - \gamma y_i \\ \frac{dz_i}{dt} = & -\delta z_i + \gamma y_i. \end{cases} \tag{1}$$

Moreover, we can rewrite the equation that describes the probability of node i being infected as

$$\frac{dy_i}{dt} = \lambda \sum_{k=1}^N \mathbf{A}_{ki} (1 - x_i - z_i) y_k - \gamma y_i \tag{2}$$

* gui.f.arruda@gmail.com

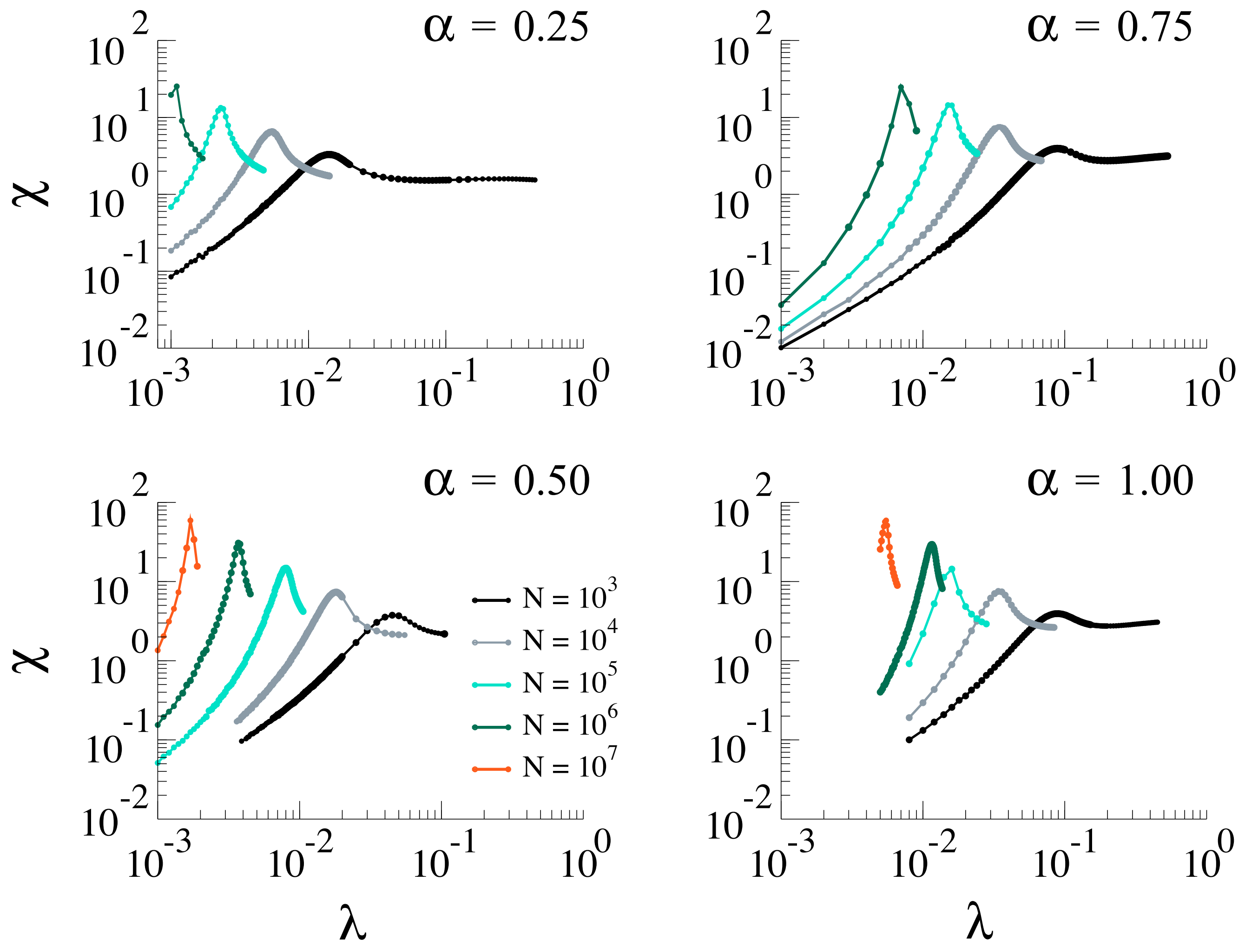

Supplementary Figure 1. Susceptibility curves for different sizes and as a function of λ for $\alpha = 0.25, 0.5, 0.75$ and 1.0, considering power-law networks, $P(k) \sim k^{-\gamma}$, with $\gamma \approx 2.25$.

Here we follow an asymptotic analysis, considering that $y_i = y_i^{(1)}\epsilon^c + O(\epsilon^{2c})$, $z_i = z_i^{(1)}\epsilon^k + O(\epsilon^{2k})$ and $x_i \in O(1)$, where $\epsilon \ll 1$. Thus, in the steady-state and neglecting the higher-order terms, we have

$$\gamma y_i^{(1)}\epsilon^c = \lambda \sum_{k=1}^{N} \mathbf{A}_{ki}(1 - y_i^{(1)}\epsilon^c - z_i^{(1)}\epsilon^k) y_k^{(1)}\epsilon^c \tag{3}$$

$$= \lambda \sum_{k=1}^{N} \mathbf{A}_{ki} y_k^{(1)}\epsilon^c + O(\epsilon^{2c}) + O(\epsilon^{ck}) \tag{4}$$

Note that the left-hand side is of order $O(\epsilon^c)$, implying that the right-hand side is also of the same order. Here, we are interested in the regime where we have a small number of recovered individuals. So, neglecting the higher-order terms we have an eigenvalue problem, yielding to the critical point given as

$$\frac{\lambda}{\gamma} = \frac{1}{\Lambda_{\max}(\mathbf{A})}. \tag{5}$$

Supplementary References

[1] S. Chatterjee and R. Durrett, Ann. Probab. **37**, 2332 (2009).

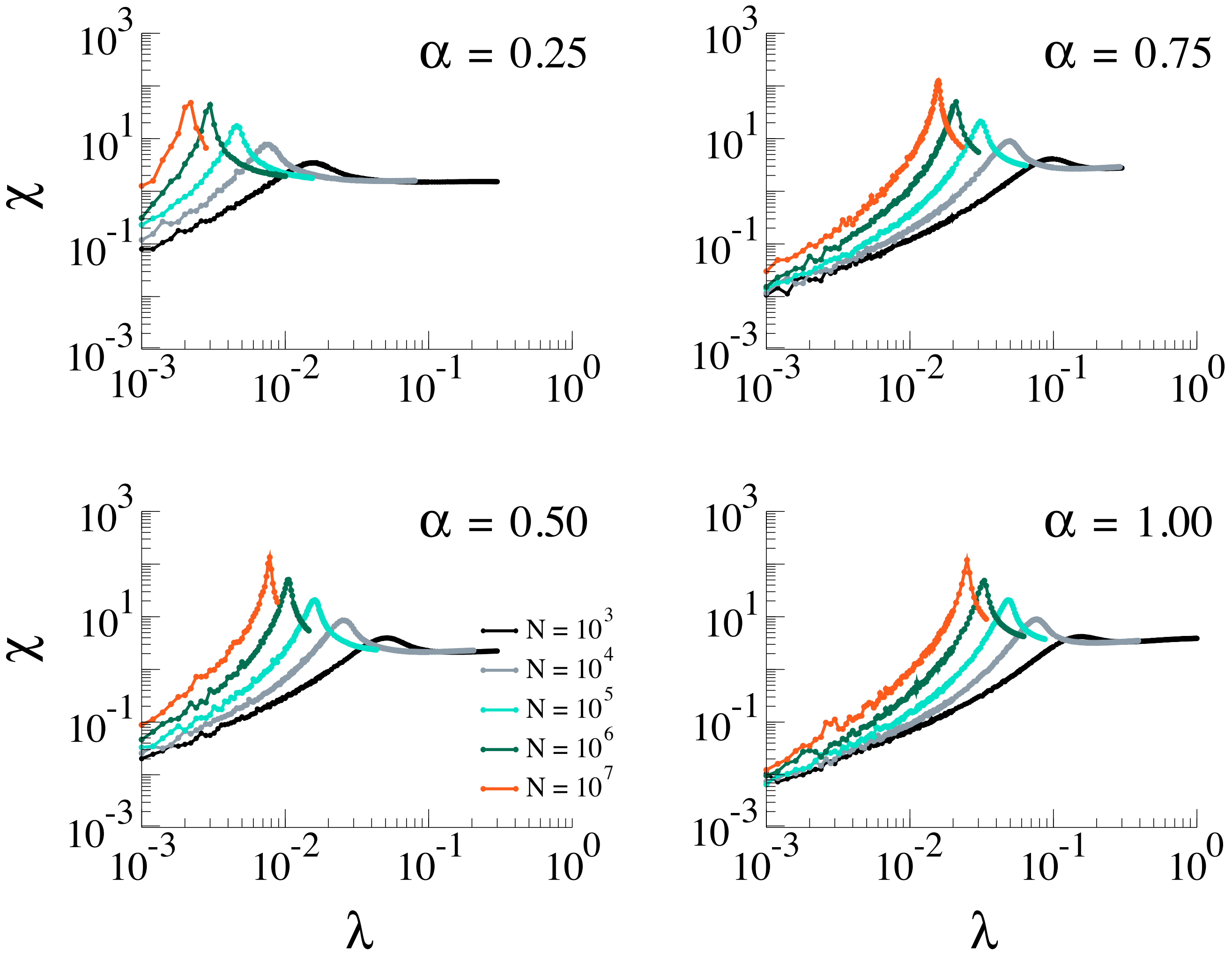

Supplementary Figure 2. Susceptibility curves for different sizes and as a function of λ for $\alpha = 0.25, 0.5, 0.75$ and 1.0, considering power-law networks, $P(k) \sim k^{-\gamma}$, with $\gamma \approx 2.75$.

[2] T. Montford, D. Valesin, and Q. Yao, Electron. J. Probab. **18**, 36 pp. (2013).
[3] G. F. de Arruda, F. A. Rodrigues, and Y. Moreno, Physics Reports **756**, 1 (2018), ISSN 0370-1573.
[4] S. C. Ferreira, R. S. Sander, and R. Pastor-Satorras, Phys. Rev. E **93**, 032314 (2016).
[5] C. Castellano and R. Pastor-Satorras, Phys. Rev. Lett. **96**, 038701 (2006).
[6] M. Karsai, R. Juhász, and F. Iglói, Phys. Rev. E **73**, 036116 (2006).
[7] W. Cota, A. S. Mata, and S. C. Ferreira, Phys. Rev. E **98**, 012310 (2018).

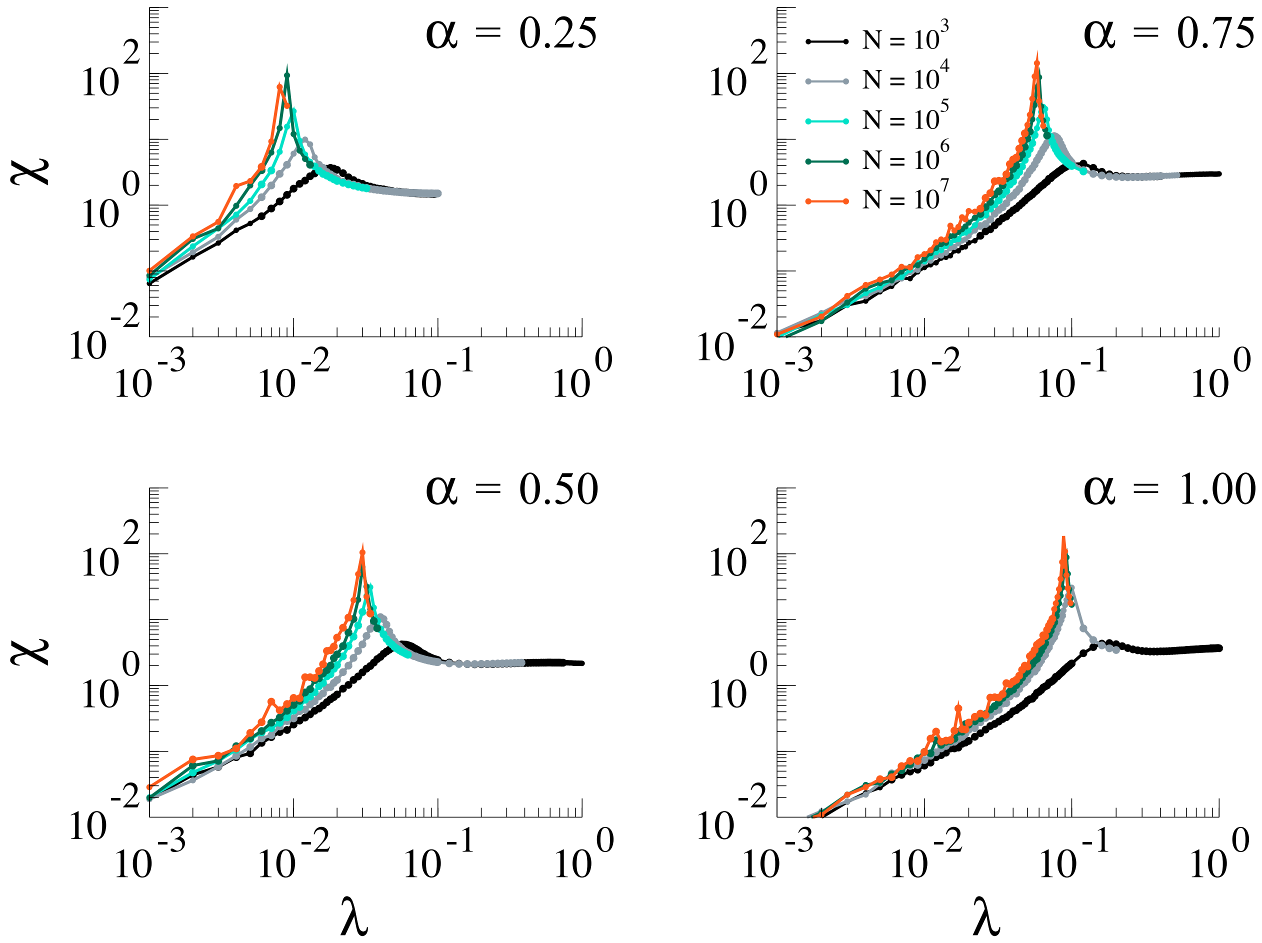

Supplementary Figure 3. Susceptibility curves for different sizes and as a function of λ for $\alpha = 0.25, 0.5, 0.75$ and 1.0, considering power-law networks, $P(k) \sim k^{-\gamma}$, with $\gamma \approx 3.5$.

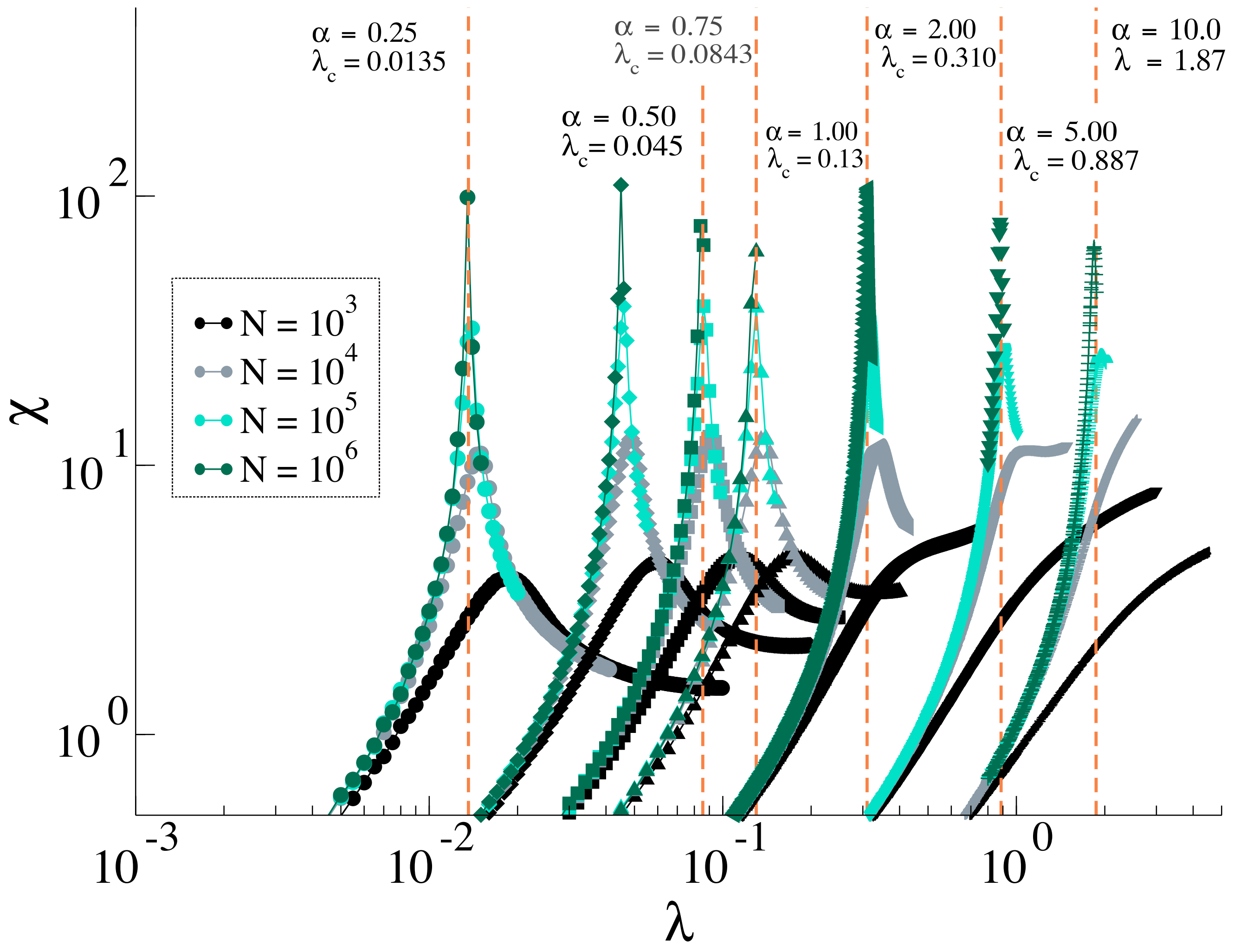

Supplementary Figure 4. Susceptibility curves as a function of λ for random regular networks with $\langle k \rangle = 10$ and different values of α and sizes.
