# Supplementary material for: From subcritical behavior to a correlation-induced transition in rumor models
Source: Nat Commun. 2022 Jun 1;13:3049. doi: 10.1038/s41467-022-30683-z (PMC9160067; doi:10.1038/s41467-022-30683-z)
Supplement: Supplementary file 3 — Description of additional Supplementary File [file 41467_2022_30683_MOESM3_ESM.pdf]

Descriptions of additional supplementary data files

Supplemental movie 1: Legend: Monte Carlo Simulations for the standard Maki-Thompson model near the critical point. File: Grid lambda critical.avi

Supplemental movie 2: Legend: Monte Carlo Simulations for the standard Maki-Thompson model at the supercritical regime. File: Grid lambda supercritical.avi
